# Oral systemic therapy: Not all “win-win”

**DOI:** 10.4103/0971-5851.68844

**Published:** 2010

**Authors:** G. S. Bhattacharyya

**Affiliations:** *Department of Medical Oncology and Clinical Hematology, AMRI Hospitals, Gariahat Road, Orchid Nursing Home, C I T Road, Scheme VI M, Phoolbagan, Kolkata, India. E-mail: docgs@hotmail.com*

The approach to cancer treatment has changed in recent years, based on research that identifies new targets and develops agents to specifically target the targets. The discovery and clinical development of such molecules has led to a new class of agents, mostly for oral administration.

Oral therapeutic agents that target receptor tyrosine kinases (e.g., imatinib, dasatinib, sorafenib, sunitinib, lapatinib) or histone deacetylases (e.g., vorinostat) were introduced in this decade. Lenalidomide, an analogue of thalido-mide, was also recently introduced. Lenalidomide and vorinostat have immunomodulatory, anti-inflam-matory, and antiangiogenic proper-ties. While standard chemotherapy drugs are usually administered intravenously and continue to be the mainstay of systemic therapy, this new and growing list of orally administered agents is rapidly gaining prominence. In fact, some of these drugs, such as imatinib in chronic myeloid leukemia and thalidomide/lenalidomide in myeloma, have become the current standards of care for these diseases. Moreover, a few classical chemotherapy drugs, such as capecitabine, etoposide, and uracil/tegafur, are also in widespread use in the management of many important cancers. Is this new trend towards orally administered anticancer drugs all for the better?

## Advantages of oral chemotherapy

Better patient convenience is the biggest ostensible advantage of orally administered drugs. The flexibility of timing and drug exposure, location of administration, and non-invasiveness are among the other advantages. Oral administration provides more prolonged drug exposure compared with intermittent intravenous infusion, which may be important for drugs with schedule-dependent efficacy. The in vivo exposure to a drug is related to concentration and time. Thus, a drug with a short half-life can achieve a greater exposure time by either continuous infusion or by continuous oral dosing. This exposure time can have profound effects on toxicity (e.g., with antifolates) or efficacy (e.g., phosphorylation).[1] The use of oral therapy has the potential to reduce the cost of healthcare resources for inpatient and ambulatory patient care services. For example, there could be less use of supplies and ancillary support personnel like nurses and techni-cians. Finally, oral therapy may be associated with a better quality of life compared to parenteral administration.

## Challenges associated with oral chemotherapy

Several potential problems arise uniquely because of the use of oral -therapy. Oncologists need to be aware of these potential problems and take steps to avoid or minimize them in order to maintain the advantages and efficacy of oral agents. Oral therapeutic agents interact with other prescription and non-prescription drugs as well as with food, nutritional supplements, and herbal remedies. Some agents (e.g., sorafenib) should not be taken with food, especially high-fat food, because the latter reduces drug absorption and bioavailability. In contrast, other agents (e.g., imatinib) should be taken with food to reduce gastrointestinal irritation. With other drugs like tamoxifen, there could be significant loss of efficacy due to interaction with other drugs like antidepressants. Dysphagia, odynophagia, nausea, and vomiting can all present as barriers to the use of the oral agents, causing missed doses or precluding treatment by the oral route. Drug absorption may also be reduced in patients who vomit within a short time after taking a dose. Malabsorption, post-gastrectomy, and diarrhea can have major effects on drug absorption.

The toxicity profiles of many newer agents differ from those of traditional chemotherapy drugs. While this allows patients to avoid some, other equally frustrating adverse effects like rashes, skin hypo- and hyperpigmentation, handfoot syndrome, hypertension, proteinuria, hypothyroidism, cardiac failure, and fluid retention have emerged in recent literature.

Non-adherence to the prescribed treatment is another potential problem with the use of oral agents at home or other non-traditional settings like assisted living facility, rehabilitation center, nursing home, or hospice. Non-adherence may be the result of confusion and misunderstanding about the treatment regimen or failure to remember doses. This problem can be confounded if the patients try to catch up on missed doses. This problem is avoided with parenteral therapy given in a clinic setting under the supervision of healthcare providers.

The counseling of patient or caregiver, which is given on oral therapy, must address the unique adverse effect profile associated with each agent. For example, life-threatening birth defects are associated with lenalidomide and thalido-mide, and male and female patients receiving these drugs are required to comply with specific requirements designed to prevent fetal exposure to the drug.

Lastly, the prohibitive cost of some of these targeted oral agents is a pressing concern that precludes their use by the majority of our patients and their possible (and inappropriate) rationing by patients who use them.

## Pharmacokinetics

The pharmacokinetic properties of the oral agents must also be considered. The ideal oral agent lacks inter-pa-tient variability (i.e., among different individuals) in absorption and area under the plasma concentration–time curve (i.e., exposure). A lack of in-tra-patient variability (i.e., over time in the same individual) in pharmacokinetics with repeated dosing (i.e., no drug accumulation) or lack of induced metabolism is also desirable. Another important ideal is the dosing algorithm. A simple basis for dosing, i.e., a flat dose that is taken by all patients every day instead of an individualized dose based on weight or body surface area, would minimize confusion and promote adherence. Ideally, a dose strength that corresponds to the flat dose would be very desirable so that patients would not need to take multiple tablets or capsules at each dose and thus minimize errors.

The pharmacokinetic properties of currently available oral agents depend on the specific agent. For example, the absorption of etoposide is saturable, resulting in lower bioavailability at large dosages compared with smaller ones. A high-fat meal decreases the absorption of some agents (e.g., sorafenib) and increases the absorption of vorinostat and other agents. In addition, oral drugs are subject to degradation in the gastrointestinal tract. The solubility of dasatinib is pH dependent, and acid suppression from proton pump inhibitors, H2-receptor antagonists, or antacids can reduce the exposure to dasatinib. Certain oral chemotherapeutic agents (e.g., etoposide, cyclophos-phamide) are subject to first-pass metabolism by intestinal and hepatic cytochrome P-450 (CYP) enzymes; particularly the 3A4 isoenzyme. The bioavailability of substrates for this isoenzyme may be reduced when the drug is administered orally compared with the parenteral route. Bioavailability also may be affected by drugs that induce or inhibit CYP 3A4.

The membrane-bound p-glycoprotein transporters, located near CYP 3A4 in the intestinal epithelium, can affect the absorption and bioavailability of chemotherapeutic agents. The gene that encodes p-glycoprotein exhibits genetic polymorphism (i.e., variability),[2] and can greatly affect intracellular exposure. Other drugs can induce or inhibit p-glycoprotein, affecting the bioavailability of oral agents. Drug activation is also an important factor.[3] Capecitabine, an oral prodrug of 5-fluorouracil (5-FU), undergoes activation through a multiple-step process. The enzyme involved in the final activation step, thymidine phosphorylase, exhibits polymorphism that can affect pharmacokinetics and patient outcomes.[4] An understanding of the pharmacokinetic variables that can affect the absorption and disposition of oral chemotherapy may allow modification of drug therapy so that patient outcomes are optimized.

## Comparative studies

The pharmacokinetics of oral chemotherapeutic agents are well characterized by the time the products are introduced, but studies comparing the clinical efficacy and safety of oral and parenteral forms of the same drug or a prodrug are less common. Oral 5-FU prodrugs and intravenous 5-FU are an exception in that they have been compared in several clinical studies. In two phase III randomized studies with a total of 1207 patients with previously untreated metastatic colorectal cancer, the response rate was 26% with oral capecitabine and 17%. With IV bolus of 5FU plus leucovorin (the Mayo Clinic regimen), a difference that is statistically significant.[5] The time to disease progression and overall survival were similar in the two treatment groups, however. A comparison of safety profiles favored capecitabine over 5-FU plus leucovorin.[6]

The efficacy and safety of oral capecitabine and intravenous bolus of 5FU plus leucovorin (the Mayo Clinic regimen) were compared over a 24-week period in the adjuvant setting in another randomized study of 1987 patients with resected stage III (Dukes’ C) colon cancer. The relapsefree survival was significantly greater in the capecitabine-treated group than in the group receiving 5-FU plus leucovorin. Capecitabine was at least as effective as 5-FU plus leucovorin in increasing disease-free survival and overall survival. Significantly fewer adverse effects were associated with capecitabine than 5-FU plus leucovorin. In the National Surgical Adjuvant Breast and Bowel Project (NSABP), oral uracil plus the 5-FU prodrug tegafur (ftorafur), a fluoro–pyrimidine combination referred to as UFT, plus leucovorin was compared with intravenous 5-FU plus leucovorin (the Roswell Park regimen) in 1608 patients with stage II or III carcinoma of the co-lon. There was no significant difference between the two treatment groups in overall survival or disease-free survival. The toxicities were similar in the two treatment groups. These studies demonstrate that use of the oral route of administration for 5-FU prodrugs instead of the intravenous route does not compromise the efficacy or safety of chemotherapy. Additional research is needed to compare outcomes when other oral chemotherapies are used instead of parenteral chemotherapy.[7]

## Patient preference

Patient preference for the route of chemotherapy administration was evaluated by interviews conducted in 103 patients with incurable cancer, who anticipated receiving palliative chemotherapy. The majority of patients (90%) preferred the oral route, primarily because of greater convenience (57%), problems with intravenous access, fear of needles (55%) or a better environment for administration of medication (i.e., the home setting). Ten patients preferred the intravenous route, and one patient had no preference for route of administration. Although most patients had a preference for one of the routes of admin-istration, a lower response rate or shorter duration of response was not an acceptable trade-off for 70 and 74% patients, respectively. Thirty-nine percent of patients wanted the decision about route of administration to be made primarily by the physician.[8]

In a randomized crossover study, 37 previously untreated patients with advanced colorectal cancer were treated with oral UFT plus leucovorin for 28 days every 5 weeks or intravenous 5-FU plus leucovorin for 5 days every 4 weeks for the first treatment cycle and then they were crossed over to the other treatment for the second treatment cycle. Twenty-seven (84%) of 32 patients completing a questionnaire preferred oral UFT over intravenous 5-FU.[9] The ability to take the medication at home, less stomatitis and diarrhea, and preference for an oral dosage form were cited by patients as the most important reasons for these preferences.[8]

## Quality of life

Studies comparing the quality of life associated with oral and intravenous chemotherapy using validated instruments are limited. The Functional Assessment of Cancer Therapy-Colorectal (FACT-C) scale, a validated tool for measuring quality of life, and a convenience-of-care analysis were used in the NSABP trial in which oral UFT plus leucovorin was compared to intravenous 5-FU plus leucovorin in patients with colon carcinoma in the adjuvant setting. There were no significant differences between the two treatment groups in FACT-C scores or overall quality of life. However, oral UFT plus leucovorin was associated with a significantly higher convenience-of-care score than intravenous 5-FU plus leucovorin.[10]

## CONCLUSION

Patients receiving chemotherapy prefer the oral route of administration over the parenteral route because of greater convenience and flexibility in the location and scheduling of medication adminis-tration. Awareness in clinicians of the potential challenges of oral drugs can help optimize patient outcomes. Additional studies comparing more oral and parenteral forms of chemotherapeutic agents are warranted.
